# Implementing Lifestyle Counselling Into Secondary Type 2 Diabetes Care: Perspectives From Diabetes Nurses

**DOI:** 10.1002/nop2.70183

**Published:** 2025-04-14

**Authors:** Chantal H. M. Pot, Eclaire A. G. Hietbrink, Gozewijn D. Laverman

**Affiliations:** ^1^ Department of Internal Medicine/Nephrology, Ziekenhuisgroep Twente Almelo the Netherlands; ^2^ Department of Biomedical Signals and Systems University of Twente Enschede the Netherlands

**Keywords:** diabetes nurse, healthcare, implementation, lifestyle, type 2 diabetes mellitus

## Abstract

**Aim:**

To explore the implementation of lifestyle counselling in secondary type 2 diabetes mellitus care from the perspective of diabetes nurses.

**Design:**

An exploratory qualitative study.

**Methods:**

Semi‐structured interviews were conducted with diabetes nurses in a hospital setting. Data were analysed through inductive thematic analysis by two independent coders.

**Results:**

The findings are structured around two main categories: the current role of lifestyle in consultations and nurses' perspectives on the future of lifestyle counselling. Diabetes nurses consider lifestyle an essential component of care but face numerous barriers to discussing it effectively. Key challenges include limited expertise, a lack of practical guidelines, time constraints, patient motivation, and the patient‐provider relationship. Nurses prioritise raising awareness, educating, and providing brief personalised advice, while comprehensive lifestyle coaching is beyond their scope. They emphasise the importance of multidisciplinary collaboration to ensure a holistic approach that incorporates multiple lifestyle factors and individual circumstances. Nurses also recognise the potential of eHealth, anti‐obesity medication, and novel insulin pumps to support lifestyle changes.

**Conclusions:**

Lifestyle counselling is complex and influenced by various factors. Efforts should focus on specialised training, actionable guidelines, and interdisciplinary collaboration. Advances in medicine and technology offer additional opportunities to enhance patient support.

**Implications for the Profession and/or Patient Care:**

The role of diabetes nurses should focus on raising awareness, providing education, and offering brief, personalised lifestyle advice. Nurses need the knowledge and skills to identify lifestyle needs and receive an overview of appropriate referral options.

**Impact:**

Training programmes should be developed to enhance nurses' competence and confidence in providing lifestyle guidance. Interdisciplinary collaboration must be strengthened both within hospitals and at the regional level. Policymakers should develop practical guidelines encompassing all lifestyle dimensions and providing clear referral pathways to specialised professionals and interventions.

**Reporting Method:**

This study adhered to the COREQ criteria.

**Patient or Public Contribution:**

No Patient or Public Contribution.

## 1 Introduction

Type 2 diabetes mellitus (T2DM) is one of the four most prevalent noncommunicable diseases worldwide, and its prevalence is rising rapidly (Zheng et al. 2018). T2DM poses increased risks for complications such as cardiovascular diseases, nephropathy, retinopathy, and neuropathy, impacting both physical health and psychosocial well‐being (Gregg et al. 2016). Additionally, T2DM significantly strains healthcare systems, society, and patients due to associated costs (Gregg et al. 2016). T2DM is largely a lifestyle‐related disease, with unhealthy behaviours being key contributors to its development (Zheng et al. 2018).

Lifestyle changes can reverse T2DM or induce remission, underscoring the need to promote healthy lifestyles in this population (Lean et al. 2019; Ried‐Larsen et al. 2019). Optimal lifestyle management involves adherence to a balanced diet rich in fruits, vegetables, whole grains, and lean proteins; engaging in at least 150 min of moderate‐vigorous physical activity per week; avoiding smoking; consuming alcohol in moderation; and ensuring sufficient relaxation (American Diabetes Association 2018). However, despite widespread awareness of its benefits, many patients struggle to adopt a healthy lifestyle due to complex personal, psychological, social, and environmental barriers (van Ommen et al. 2017). Moreover, adherence to lifestyle advice remains low (Jalving et al. 2018), limiting optimal T2DM management.

International guidelines for T2DM management emphasise the importance of lifestyle counselling as a cornerstone of effective care. Recommendations from organisations such as the “American Diabetes Association” and the “European Association for the Study of Diabetes” underscore the critical role of a healthy lifestyle in individuals with T2DM (American Diabetes Association 2018; Aas et al. 2023). With rapid developments in lifestyle medicine, such as lifestyle intervention programs (van Rinsum et al. 2018) and eHealth technologies (Obro et al. 2021), the role of healthcare providers in facilitating lifestyle changes is evolving. This transformation further underscores the need to examine how these developments may affect the support patients receive, particularly from nurses who are often on the frontline of patient education.

## 2 Background

Nurses are uniquely positioned to bridge the gap between clinical guidelines and patient behaviour in the management of T2DM. Their role extends beyond medication management to include tailored education, ongoing support, and culturally sensitive interventions that foster sustainable lifestyle changes (van Dillen and Hiddink 2014). In the Netherlands, patients with complex T2DM or additional comorbid conditions are referred to secondary care hospital settings, where healthy lifestyle practices remain a cornerstone of treatment. Secondary care diabetes nurses and nurse practitioners (NPs) play a crucial role in managing various aspects of diabetes (Eerste Associatie voor Diabetesverpleegkundigen 2011). Diabetes nurses complete a specialised post‐bachelor training that equips them with the competencies needed for the broad scope of T2DM management, including tailored lifestyle counselling. Although clinicians retain the final medical responsibility, much of the day‐to‐day management and patient support is provided by diabetes nurses. NPs have an advanced clinical role with prescribing authority and an independent treatment relationship. Like diabetes nurses, they also play a key role in lifestyle counselling.

Studies in other settings have demonstrated that the comprehensive integration of lifestyle counselling into routine practice can be further improved (Stuij et al. 2017; Brotons et al. 2012). Common challenges reported by healthcare providers include limited consultation time, a lack of specific expertise in lifestyle medicine, and difficulties in addressing patient motivation and knowledge (Hébert et al. 2012; Stuij 2018; Nauta et al. 2022). Several studies conducted among nurses have confirmed that insufficient training, limited competencies, restricted consultation time, and low patient motivation are significant barriers to effective lifestyle counselling (Jansink et al. 2010; Bräutigam Ewe et al. 2021; Behm et al. 2024; Oikarinen et al. 2024). Additionally, the level of organisational support also plays a critical role in the success of lifestyle counselling practices (Behm et al. 2024; Oikarinen et al. 2024).

Although several studies have explored the role of lifestyle in healthcare, no studies have specifically focused on the perspectives of diabetes nurses and NPs in secondary care. Managing T2DM in this context may involve specific experiences and sensitivities regarding lifestyle counselling that warrant further investigation (Oikarinen et al. 2024). While previous research has primarily identified barriers and facilitators, little is known about how diabetes nurses themselves envision effective lifestyle counselling, particularly considering recent developments in lifestyle medicine. A deeper understanding of these challenges and opportunities may provide valuable insights from a nursing perspective, identifying the necessary elements to achieve optimal lifestyle counselling.

## 3 The Study

The study aims to address the gaps by exploring diabetes nurses' current practices, the barriers they encounter, and their future perspectives regarding lifestyle promotion in daily practice. The research objectives are as follows:

1. To gain insights into the extent to which lifestyle is discussed during consultations with diabetes nurses.
2. To investigate the main barriers and facilitators for discussing lifestyle during consultations.
3. To explore the views of diabetes nurses regarding the implementation of lifestyle in consultations and future care.

## 4 Methods

### 4.1 Design

This study uses an exploratory qualitative design with semi‐structured interviews. We chose a qualitative approach to gain a deeper understanding of diabetes nurses' perceptions regarding the role of lifestyle in standard care.

### 4.2 Theoretical Framework

We used the domains from the Consolidated Framework for Implementation Research (CFIR) (Damschroder et al. 2022) to guide the development of our interview guide. CFIR is a widely used implementation framework that facilitates a systematic assessment of barriers and facilitators in the development of tailored implementation strategies. It is one of the most frequently applied frameworks for evaluating the implementation of clinical practice guidelines in healthcare (de Freitas Mello et al. 2024). In nursing research, CFIR has been utilised to examine implementation challenges, demonstrating its relevance and applicability in this field (Breimaier et al. 2015). The CFIR comprises five domains: intervention characteristics, outer setting, inner setting, characteristics of individuals, and process of implementation. Using the CFIR, we ensured that the interview aligned with the domains influencing the implementation of a novel approach or innovation. We aimed for a comprehensive understanding of factors affecting the implementation of lifestyle in healthcare.

### 4.3 Study Setting and Recruitment

The participants were diabetes nurses and NPs within the Department of Internal Medicine at Ziekenhuisgroep Twente (ZGT), a regional hospital in the Netherlands. ZGT has a diabetes outpatient clinic where internists, NPs, and diabetes nurses provide daily care for a large population of T2DM patients. Diabetes professionals in the Netherlands follow national evidence‐based guidelines that emphasise the vital role of lifestyle counselling in diabetes management. These include the guidelines of the Dutch Association of Internal Medicine (NIV), the Dutch College of General Practitioners (NHG), and the Dutch Diabetes Federation (NDF). All guidelines highlight the importance of patient education on various lifestyle behaviours and weight management. When further individual guidance is needed, referral to specialised healthcare professionals, such as dietitians, physiotherapists, or psychologists, is recommended. Diabetes nurses and NPs incorporate lifestyle advice into nurse–patient consultations, tailoring it to each patient's needs and readiness for change. Diabetes nurses conduct individual consultations, develop personalised care plans, educate patients about diabetes management, and monitor and adjust treatment regimens in cooperation with the physician. In addition to diabetes nurses, NPs specialised in diabetes care also play a key role in lifestyle counselling. While NPs have an independent treatment relationship with patients, their responsibilities in providing tailored lifestyle counselling are comparable to those of diabetes nurses. Compared to treating internists, diabetes nurses and NPs have more frequent contact with T2DM patients and more time per consultation. ZGT employs seven diabetes nurses and one clinical NP specialised in diabetes (*N* = 8) eligible for participation in this interview. All eligible participants were recruited based on purposive sampling during monthly meetings attended by the nurses. Information about the study was provided to participants via email, and appointments were scheduled accordingly. All nurses who were approached were willing to participate in the study.

### 4.4 Inclusion and/or Exclusion Criteria

Participants were required to have completed their training as a diabetes nurse or NP specialised in diabetes and must conduct consultations with people with T2DM. No exclusion criteria were formulated.

### 4.5 Data Collection

The interviews took place from early December 2023 to late January 2024 at ZGT Hospital. Each interview was conducted in person, in the nurse's consultation room. The interviews ranged from 21 to 39 min, with an average duration of 30 min. All interviews were audio recorded.

The interviews were structured to assess the current role of lifestyle in consultations, facilitating and hindering factors for lifestyle discussions, and future perspectives regarding lifestyle in healthcare (Appendix S1).

The interview began with a general introduction about the study's purpose, followed by reviewing and signing the informed consent form. To gain insights into participants' perceptions of lifestyle, the interview began with a general introductory question asking participants how they define lifestyle. The topic “current situation” aimed to understand the structure and key discussion points of diabetes nurse–patient consultations and the role of lifestyle within them. Under the topic “facilitating and hindering factors for lifestyle discussion”, questions were formulated to explore factors promoting or obstructing the discussion of lifestyle during consultations. The final topic, “future vision”, aimed to reveal diabetes nurses' perspectives on lifestyle in healthcare in the future. For each topic, one or two open‐ended questions were formulated to elicit the views of the diabetes nurses. Examples of interview questions included the following: “What is the role of lifestyle in your consultations?”, “What factors influence the discussion about lifestyle in people with type 2 diabetes?”, and “What is your vision regarding lifestyle management in type 2 diabetes care in the future?”. Based on participants' responses, follow‐up questions were asked to explore their perspectives in greater depth. This was facilitated by probing and prompting questions for each topic, allowing for follow‐up questions as needed. The interview concluded with a question about any unaddressed themes or additional questions or comments from the participant. The full interview schedule is available in Appendix S1.

A digital questionnaire aimed to collect background characteristics, including gender (male/female/other), age (categorised by years), role (diabetes nurse/nursing specialist/other), and years of experience in the current role (categorised by years).

### 4.6 Data Analysis

The interviews were digitally recorded and transcribed non‐verbatim using artificial intelligence via Good Tape Transcription software. These transcripts were then reviewed and adjusted by the researchers based on the audio files.

The transcripts were analysed using inductive thematic analysis (Braun and Clarke 2006) with ATLAS.ti version 23. This method involves systematically identifying and analysing themes within the data. Each interview was independently double‐coded by two coders (C.H.M.P. and E.A.G.H.). Text fragments that appeared to offer important perspectives on one of the research questions were assigned descriptive, open codes. Any discrepancies in the assigned open codes were discussed and resolved. The open codes were then analysed and structured through axial coding, where codes were compared and merged into overarching codes serving as sub‐themes. These axial codes formed the basis for developing, defining, and refining the themes. Throughout this process, the overarching codes were regularly reviewed and adjusted until consensus was reached within the research team.

Background characteristics obtained from the questionnaire were reported as frequency (percentage) because all variables were categorical.

### 4.7 Ethical Considerations

Approval was obtained from the review committee of the University of Twente (number 220569). The study was conducted in accordance with the Declaration of Helsinki. All participants provided written informed consent. All data identifiable to participants were removed or pseudonymized. The audio recordings were deleted after transcription. The data are stored on a secure, encrypted server at ZGT, accessible only to the researchers.

### 4.8 Rigour and Reflexivity

To ensure the rigour of the study, we used a predefined, transparent methodology grounded in the CFIR and guided by the Consolidated Criteria for Reporting Qualitative Research (COREQ). In this exploratory study, we focused on the concept of information power rather than data saturation to guide our sample size (Malterud et al. 2016). Our aim was not to provide a complete description of all aspects of the subject but rather to offer new insights that challenge or contribute to current knowledge. Therefore, we determined that a smaller yet relevant sample of diabetes nurses would be sufficient. Data analysis was performed by two authors, with a third author involved in validating the findings. After the analysis, the results were presented to participants for member checking, and they confirmed that the findings reflected their views on lifestyle in diabetes care.

In terms of reflexivity, all interviews were conducted by one female interviewer (C.H.M.P.), a master's student in medicine with specialised knowledge and interest in lifestyle medicine. The interviewer followed a pre‐established interview guide and had prior experience in conducting interviews, complemented by further study in interview techniques before data collection. Importantly, the interviewer had no prior relationship with the participants, which ensured neutrality. The interviewer introduced herself and the aims of the study before the interview. Before the interviews, the interviewer observed several consultations with diabetes nurses. This approach allowed for a deeper understanding of the context and structure of the consultations, which contributed to a more informed interpretation of participants' responses.

## 5 Results

### 5.1 Characteristics of the Participants

All participants were female, consisting of seven diabetes nurses and one NP (Table 1). Three participants were between 30 and 44 years old, three between 45 and 59 years, and two participants were 60 years or older. Two participants had 2–5 years of experience in their role, two had 6–10 years of experience, and four had more than 10 years of experience.

**TABLE 1:** Participant characteristics (*N* = 8).

Characteristics	*N* (%)
Gender	
Female	8 (100)
Male	0 (0)
Other	0 (0)
Age (years)	
18–29	0 (0)
30–44	3 (37.5)
45–59	3 (37.5)
60 and older	2 (25.0)
Profession	
Diabetes nurse	7 (87.5)
Nurse practitioner diabetes	1 (12.5)
Other	0 (0)
Number of years of experience as diabetes nurse/nurse practitioner in the hospital (years)	
0–5	2 (25.0)
6–10	2 (25.0)
More than 10	4 (50.0)

### 5.2 Current Practices in Lifestyle Discussions During Consultations

The first category of findings addresses the current situation and the factors influencing the lifestyle discussion according to the participants. The identified main and sub‐themes are presented in Table 2.

**TABLE 2:** Perceptions about lifestyle, the nurse–patient consultation, and lifestyle conversations.

Themes and sub‐themes	Definition
Perceptions regarding lifestyle	
Definitions of lifestyle	Meaning of lifestyle according to the diabetes nurses
Importance of a healthy lifestyle	Perceptions regarding the importance of a healthy lifestyle for prevention and diabetes management according to diabetes nurses
Complexity of lifestyle and lifestyle changes	Views on the complexity of lifestyle change in individuals with diabetes
Characteristics of the nurse–patient consultation	
Consult structure and format	Insights into the preparation, structure, and format of the consultation by diabetes nurses
Patient‐centered approach	Opinions on the importance of tailoring the consultation to individual needs
The role of lifestyle in the nurse–patient consultation	
Diversity in lifestyle guidance among diabetes nurses	Insights into the approach to lifestyle discussions with people with T2DM by diabetes nurses
Personalised lifestyle approach	Insights into the personalised approach to lifestyle in the consultation
Outsourcing lifestyle expertise	Insights into involving the expertise of other healthcare professionals for professional lifestyle guidance and treatment
Current use of the Electronic Health Record (EHR)	Insights into using the EHR for documentation about the lifestyle of the patient
Use of eHealth	Insights into using eHealth to support the lifestyle discussion
Factors influencing lifestyle discussions	
Expertise and competencies of diabetes nurses	Insufficient knowledge among diabetes nurses about healthy lifestyle and guiding lifestyle
Applicability of literature and guidelines in practice	Guidelines and literature regarding lifestyle are difficult to apply in daily practice
Time	Insufficient time within the consultation for discussing lifestyle
Relation with the patient	The importance of a good patient relationship for conducting the lifestyle discussion
Patient knowledge and motivation	Lack of knowledge and motivation among patients for lifestyle changes

#### 5.2.1 Perceptions Regarding Lifestyle

This main theme relates to the perceptions of participants regarding lifestyle. Defining lifestyle yielded a variety of responses, with nutrition and physical activity mentioned universally. Other mentioned aspects included sleep, stress, relaxation, smoking, meaning, and glucose regulation. The multifaceted nature of lifestyle was articulated by one of the participants as follows:

Yes, lifestyle is everything. Lifestyle also includes sleep, which is important. Lifestyle is being physically active, eating healthily, being social… I think lifestyle is not just about nutrition but about the whole picture. And for people with diabetes, it is about trying to avoid spikes in blood sugar levels. I believe people with diabetes have a lot on their plate. Often, they are overweight, on insulin and are trying to lose weight, which leads to low glucose levels which causes them to feel poorly, which they then fix by eating again. I am mainly talking about type 2. … “So, lifestyle. It is challenging, incredibly challenging. People often have stress, sleep poorly, and eat differently because of it. It is the whole package.” (Participant 3)

All participants emphasised the importance of lifestyle in preventing and treating T2DM. They highlighted its benefits, including improved well‐being, the potential to reduce or discontinue diabetes medication, and a lower risk of complications and hospitalisations. Participants emphasised that lifestyle changes are complex because different lifestyle factors influence one another, making their integration into healthcare challenging. Specifically, they mentioned challenges related to overweight, the influence of the living environment, and the interactions between medication use and weight management.

#### 5.2.2 Characteristics of the Nurse–Patient Consultation

This main theme refers to the structure and characteristics of the nurse–patient consultation. All participants addressed essential components in the consultation, such as discussing lab results, glucose levels, medication, and conducting physical examinations (e.g., weight, blood pressure, and foot check). The sequence and content of these components vary among participants, tailored to their personal routines and patient needs. Consultations often begin with a general question about the patient's well‐being. Some participants then use medical results as a basis for discussing other topics, such as mental well‐being or lifestyle. Several participants felt that a fixed structure and preparation for the consultation lead to a more effective conversation.

A patient‐centered approach is crucial; the consultation is tailored to the specific needs of the patient with a focus on shared decision‐making and promoting the patient's self‐efficacy. This includes thoroughly exploring the patient's perspectives and reinforcing their role in managing their condition.

#### 5.2.3 The Role of Lifestyle in the Nurse–Patient Consultation

This main theme focuses on the role of lifestyle in nurse–patient consultations. Participants tailor their lifestyle discussions based on individual patient needs, with a primary focus on nutrition and exercise, supplemented by aspects like sleep and stress.

Most participants viewed lifestyle guidance as offering brief advice on healthy choices, such as eating whole grains, reading food labels, and staying active. Participants encourage patients to set small, achievable goals and recognise successes to boost motivation. Regular evaluation of lifestyle changes during follow‐up consultations is common for some participants. The emphasis is on a non‐judgmental, patient‐centred approach using shared decision‐making. Referrals to dietitians, lifestyle coaches, psychologists, and combined lifestyle interventions (CLIs) are made to support more complex lifestyle adjustments and interventions.

Notes on the lifestyle of the patient are recorded by some participants in the Electronic Health Record (EHR) without a standardised approach. However, several participants mentioned not using or being unaware of the existing lifestyle format in the EHR.

Participants acknowledged the potential role of eHealth applications like apps and wearables in providing insights into patients' lifestyles. Although not yet widely used, some participants integrate data from these technologies, such as Fitbit or Freestyle Libre, to monitor glucose regulation and support lifestyle discussions. The use of eHealth in consultations was described by one participant as follows:

I try to remain close to the patient and make small lifestyle adjustments. That is important. Sometimes, if someone [the patient] already wears a smartwatch, I ask, have you [the patient] ever noticed that there is a step counter on it? Do you ever consciously look at it? Often they do not. I tell them to take a look. What are your daily steps, could you increase them? In that way, yes. It comes up in almost every conversation, something about lifestyle. (Participant 3)

#### 5.2.4 Factors Influencing Lifestyle Discussions

This main theme explores the factors influencing lifestyle discussions. One of these factors is the lack of in‐depth knowledge and specialised training among diabetes nurses, making them feel limited in their ability to provide lifestyle guidance. Furthermore, many participants experienced difficulties with the applicability of literature and guidelines, which are often not straightforward or directly applicable in practice. This results in confusion and inconsistent advice to patients, as highlighted by a participant emphasising the variation in dietary advice:

What I find challenging … What is healthy, where should you really guide people [T2DM patients] towards? One says low‐fat yogurt, another says full‐fat products [information sources]. What is good for your cholesterol and what is not. I think people [patients] receive so much conflicting advice. Also, from doctors or from us. … “So, what is missing? Yes, a good guideline but one that is also evidence‐based. The Dutch nutrition center recommends eating 6 to 8 slices of bread but I find that challenging for people with diabetes. Sometimes I find the Dutch nutrition center quite distant from us.” … “And there are also people, those diabetes nurses and dietitians who heavily promote the low‐carb lifestyle. But I think science itself does not know what is healthy and what is not. And all the nutrition scientists contradict each other.” (Participant 3)

Additionally, time constraints were seen as a limiting factor by several participants; the available time during consultations is often insufficient to adequately address lifestyle alongside other responsibilities. A good relationship with the patient was considered fundamental for conducting lifestyle discussions. This relationship must be carefully built before space and trust are created for discussing lifestyle aspects. Participant 7 said,

And what is most important? A good relationship with patients, that is key. Even if their diabetes is not well‐managed, that does not matter. I also believe that, even if someone does not take action on their diabetes, it is important that they keep coming back to you. You need to keep the door open. So, never be judgmental. Trust is the most important thing. (Participant 7)

Diabetes nurses considered patient knowledge and motivation crucial for successful lifestyle changes. Without patient engagement, implementation becomes challenging. Participants noted that a lack of motivation and sense of responsibility made lifestyle discussions difficult. As a result, some nurses chose not to push the topic or revisited it only in follow‐up consultations if the patient was unwilling to change. One participant described this point as follows:

Besides, I think it is always a consideration—how far should you go in chasing after [unmotivated patients]? You still feel a certain duty of care. But on the other hand, should you spend your time on those who are actually willing to make changes and where you can see progress? So, it is always a bit of a balancing act, I think. (Participant 6)

Additionally, all participants believed that the responsibility for lifestyle changes lies with the patient. While the diabetes nurse can assist in identifying motivation and provide tools for lifestyle changes, they aim to return the responsibility for implementing these changes back to the patient.

### 5.3 The Vision on Lifestyle in the Consultation and Care in the Future

The participants' future visions regarding lifestyle in consultation and healthcare have been formulated. The identified main and sub‐themes and their corresponding definitions are presented in Table 3.

**TABLE 3:** Visions regarding lifestyle in future.

Themes and sub‐themes	Definition
Integration of lifestyle into consultations	
Role of the diabetes nurse regarding lifestyle	Perceptions about lifestyle discussion within the role and tasks of the diabetes nurse
Needs regarding time investment for lifestyle in the consultation	Needs regarding the time spent for discussing lifestyle
Need for expertise development of healthcare professionals	Needs for knowledge acquisition regarding lifestyle discussion and interventions
Added value of eHealth in the conversation	Perceptions about the possible added value of eHealth in the conversation about lifestyle
Future role of the Electronic Health Record (EHR)	Vision regarding the use of the EPD for standardising and structuring the lifestyle discussion
Integration of lifestyle into healthcare	
Integrated care and collaboration	Perceptions regarding collaboration and integration of care within different care settings and specialties
Lifestyle support tailored to the individual	Opinions of diabetes nurses on tailoring lifestyle support to the individual
Lifestyle guidance provided by professionals	Views on providing intensive guidance by professional in the field of lifestyle
Support for lifestyle change for the patient	
Support from anti‐obesity medication, bariatrics, and medical technology	Opinions on the use of anti‐obesity medication, bariatrics, and medical technology that support lifestyle changes and weight loss for patients
Support from eHealth	Perceptions regarding the role of eHealth in supporting lifestyle changes for patients

#### 5.3.1 Integration of Lifestyle Into Consultations

This main theme explores participants' future visions regarding lifestyle discussions during consultations. The emphasis was on the necessity of consistently integrating lifestyle into consultations. While several participants stated that they were not experts in lifestyle guidance and that they were not specifically trained for this role, they supported the idea of providing brief lifestyle education and advice. However, multiple participants felt that intensive lifestyle guidance was not part of their role partly due to the many other tasks they had related to diabetes management. The nurses saw a role for specialised lifestyle professionals or programs, such as dietitians or CLIs. One participant stated,

Well, I think basic skills are important, explaining soft drinks and sweeteners, healthy nutrition, and getting enough exercise. But going deeper? That is really the role of a dietitian, who can work with the patient to assess their energy needs and find ways to adjust their diet without causing deficiencies. … “I do not feel trained for that, and I do not think I need to be, that is what dietitians are for.” (Participant 4)

While some participants already take extra time or feel the need for additional time for lifestyle guidance, others consider this unnecessary. One participant stated:

No, but then the consultations would become too long. No, I think it is fine as it is. What matters to me is that patients receive information about lifestyle, that is key. Especially for type 2 diabetes, because that is what we are talking about—lifestyle is definitely responsible for around seventy percent of the problems, maybe even more. But if there was someone that patients could see alongside us, that would be very beneficial. I just do not feel the need to take on that role myself. (Participant 8)

Several participants expressed a desire to expand their knowledge and skills in the field of lifestyle to be better equipped to provide lifestyle advice. Some nurses were already actively deepening their understanding of lifestyle through symposia, books, and podcasts. Others mentioned the need to offer knowledge enhancement within the hospital or region, such as lifestyle‐focused education and sharing the latest developments during work meetings.

eHealth can play a supportive role in conversations about lifestyle. Participants believed that eHealth provides insight into their patients' lifestyles, using the data as a gateway to discuss lifestyle topics. This allows for setting more specific goals and offering personalised advice according to the participants. However, its integration into the healthcare process should not incur additional time. One of the participants said the following:

For example, take the second patient we saw this morning. He happens to be part of a study that allows him to use a Fitbit, measure his glucose values with the Freestyle Libre, and track his nutrition with an app. That gives you much more insight into what someone is actually doing. His glucose levels were very high, his HbA1c was way too high, so the first thought is, ‘Oh, he must be eating a lot or not exercising enough.’ But in reality, he was very active—probably even more than I am during the week. So clearly, the issue was not there, and we had to look elsewhere. That kind of tracking makes it easier to pinpoint where the real problem lies for each patient. (Participant 6)

It was also suggested to include a lifestyle overview in the EHR to discuss lifestyle aspects in a structured and effective manner. Suggestions for this included integrating a lifestyle section in the notes and adding an overview of lifestyle parameters on the cover page of the file.

#### 5.3.2 Integration of Lifestyle Into Healthcare

This main theme consists of the participants' future visions regarding the integration of lifestyle into healthcare. The participants believed improved collaboration within the hospital and region is essential for effective lifestyle guidance. Many participants emphasised that lifestyle guidance should mainly take place in primary care, with an emphasis on prevention and information provision about lifestyle at the moment of diagnosis. Examples of potential initiatives for collaboration within the hospital include organising lifestyle symposia, developing informational brochures, walking groups, and offering lifestyle programmes. The vision was that such programmes strengthen collaboration within and between healthcare teams. One of the participants says,

We have a nice department at the obesity center. But it is now mostly focused on gastric bypasses. And it would be great if it becomes an obesity center where people can also work on their lifestyle. So that they can lose weight without surgery. There are multiple ways to work on obesity. And it should also be possible within ZGT to exercise more. Or, well, to have more programs to lose weight. And not just a gastric bypass. … “Yes, and we [the diabetes department] could also connect to that. Because then the glucose levels will drop. And it [lifestyle care] becomes more collaborative.” (Participant 5)

Additionally, all participants supported a future lifestyle approach that is tailored to individual patient preferences and needs, emphasising personalised information and a broader focus beyond diabetes alone. Lifestyle advice should be adapted to factors such as the stage of diabetes, patient motivation, lifestyle preferences, and financial resources.

Participants saw a key role for professionals specialised in lifestyle, such as lifestyle coaches or dietitians, to provide in‐depth support, transcending healthcare settings and disciplines. To achieve this, healthcare professionals need to be well informed about referral options for lifestyle guidance and to monitor the progress during the consultation; feedback from the lifestyle professionals to the diabetes nurses is necessary.

#### 5.3.3 Support for Lifestyle Change for the Patient

Several participants emphasised the importance of anti‐obesity medication, bariatric surgery, and technological innovations in supporting lifestyle changes and weight loss. Specific examples included GLP‐1 agonists and advanced insulin pumps. One of the participants shared the following about the new developments:

It is amazing to see all the developments that are happening. Many old medications such as insulin, made people gain weight, but now, there are medications that actually help with weight loss, which is great for us to see. There is also modern technology, such as the 780G pumps and closed‐loop systems. These advancements help us to gain more control over diabetes, which motivates us further, and also encourages patients to actively manage their condition. (Participant 2)

Participants further indicated that eHealth could support patients in changing their lifestyles. According to them, eHealth can provide insights into patients' lifestyles, motivating and encouraging them to take proactive steps toward lifestyle improvements. Additionally, it can offer insights into the effects of lifestyle changes on health parameters, such as glucose levels. Several participants emphasised the importance of eHealth being user‐friendly for successful implementation.

## 6 Discussion

In this study, we conducted qualitative research on the implementation of lifestyle into standard diabetes care. In current practice, lifestyle is considered an important topic, but approaches vary widely among nurses. A personalised approach is seen as crucial. Several barriers hinder effective lifestyle guidance, including time constraints, impractical guidelines, limited expertise, and low patient motivation. Regarding future perspectives, diabetes nurses see lifestyle as essential for future consultations, emphasising awareness raising and providing brief lifestyle advice. Anti‐obesity medications and modern technologies can play a supportive role in this. While they consider lifestyle guidance important, they do not see it as their primary role and believe that intensive lifestyle guidance should be outsourced to experts. They stress the need for further training in order for them to be able to provide lifestyle advice confidently and emphasise the need for interdisciplinary collaboration for improvements across all these areas.

### 6.1 Principal Findings

The results indicate that diabetes nurses perceive lifestyle as a complex and broad concept. It was emphasised that lifestyle encompasses various interconnected aspects, with diet and exercise being the most discussed factors. There is no uniform approach to lifestyle guidance. Over recent years, lifestyle has evolved into an essential part of care, as recognised by the diabetes nurses in this study and T2DM guidelines (American Diabetes Association 2018; Federatie Medisch Specialisten 2025). However, the practical application of lifestyle in consultations still appears to be a challenge.

Our findings reflect common international challenges in integrating lifestyle counselling into diabetes care (e.g. Nauta et al. 2022; Jansink et al. 2010; Bräutigam Ewe et al. 2021; Behm et al. 2024; Oikarinen et al. 2024). These barriers include (1) the knowledge and expertise of the healthcare provider, (2) the applicability of guidelines in practice, (3) the amount of available time, (4) patient motivation for lifestyle changes, and (5) the patient‐healthcare provider relationship. These findings suggest that the challenges surrounding lifestyle care are universal and not unique to lifestyle counselling T2DM patients in a secondary care setting. This underscores the value of interdisciplinary collaboration to address these barriers.

A significant barrier to discussing lifestyle is the lack of practical, applicable literature and guidelines, leading to uncertainty among diabetes nurses. Scientific literature does not offer consistent lifestyle advice and guidelines lack practical tools and resources that can be directly applied in consultations (Jansink et al. 2010; Bräutigam Ewe et al. 2021; Behm et al. 2024). While Dutch guidelines for T2DM management emphasise the importance of lifestyle counselling (Federatie Medisch Specialisten 2025), they also lack practical application according to the diabetes nurses. This creates a gap between evidence‐based recommendations and real‐world implementation. Diabetes nurses in our study expressed a need for practical guidelines and tools to enhance their ability to provide lifestyle counselling. Guidelines should cover a broader range of lifestyle factors beyond diet, physical activity, and smoking, such as stress management and sleep. Additionally, clearer guidance on available community‐based lifestyle interventions and referral pathways would help professionals direct patients toward appropriate support more effectively (Te Loo et al. 2024).

Patient motivation and relationship are two factors influencing lifestyle discussions, also highlighted in other studies (Stuij 2018; Jansink et al. 2010; Behm et al. 2024). Several studies indicate that a strong patient relationship and enhancing patient self‐efficacy act as facilitators in lifestyle support, leading to more willing patients and motivated healthcare providers (Nauta et al. 2022; Behm et al. 2024). A lack of knowledge and motivation among patients reduces the motivation of diabetes nurses to guide them, despite feeling a duty of care and professional responsibility. Motivational interviewing is recognised as an effective approach in implementing lifestyle recommendations, promoting behaviour change by enhancing intrinsic motivation and resolving ambivalence (Van Wormer and Boucher 2004). This patient‐centered approach enhances self‐efficacy and strengthens the patient‐provider relationship. Training in this technique could potentially contribute to the successful implementation of lifestyle discussions.

The findings of this study emphasise the need for continuous education and knowledge development among healthcare providers regarding lifestyle guidance. The growing recognition of lifestyle medicine in the Netherlands offers promising opportunities for enhancing healthcare providers' expertise in lifestyle counselling. Organisations such as the “Dutch Coalition for Lifestyle in Healthcare” and the “Association for Physicians and Lifestyle” actively promote lifestyle integration into medical practice, emphasising education and professional training (Coalitie Leefstijl in De Zorg 2025). As these initiatives evolve, diabetes nurses and other healthcare professionals may become better equipped to provide effective lifestyle counselling in diabetes care.

The vision of diabetes nurses regarding the future of lifestyle guidance varies. Most nurses do not see intensive lifestyle guidance as their responsibility. Diabetes nurses advocate for an interdisciplinary approach, suggesting that the basics of lifestyle guidance, such as awareness raising, education, and brief personalised advice, remain part of their role. However, they propose that more specialised guidance should be left to other disciplines. The findings also align with the expressed need for increased multidisciplinary collaboration.

Many diabetes nurses believe that intensive lifestyle guidance should be provided in primary care. However, similar barriers to providing lifestyle guidance are experienced in primary care (Hébert et al. 2012; Jansink et al. 2010; Bräutigam Ewe et al. 2021). In the Netherlands, both primary and secondary care are increasingly integrating lifestyle interventions to provide more structured support for patients (Te Loo et al. 2024). However, regional collaboration between primary and secondary care remains limited, which may affect the feasibility of a multidisciplinary approach, as envisioned by diabetes nurses. The hospital where this study was conducted operates within a regional healthcare network where collaboration on lifestyle interventions is still developing. Future research could explore how these findings compare across different Dutch hospitals and other settings to better understand how organisational structures influence lifestyle counselling.

To address the need for structured lifestyle interventions, the Netherlands has implemented several national programmes that could support diabetes nurses in guiding patients. The combined lifestyle intervention (CLI) (van Rinsum et al. 2018) is covered by basic health insurance for individuals with overweight and T2DM, offering intensive lifestyle coaching across multiple domains. Additionally, some hospitals, including one in our study region, have introduced Lifestyle Front Offices (LFOs) (van Dijk et al. 2023). LFOs are centralised support desks where trained professionals refer patients toward community‐based lifestyle change initiatives. These initiatives represent a unique opportunity to bridge the gap between secondary care and long‐term lifestyle support. Additionally, they counteract the fragmentation of lifestyle guidance and offer an integrated approach to lifestyle that considers the whole person. CLIs and LFOs have the time and expertise to take a holistic approach, identifying the complex personal, cultural, social, and environmental barriers that influence lifestyle behaviours (van Dijk et al. 2023). By addressing these factors, they can provide more tailored, patient‐centred interventions. Diabetes nurses can play a crucial role in these initiatives by motivating patients and ensuring appropriate referrals to CLIs or LFOs. Strengthening their involvement in such programmes could help standardise lifestyle counselling in secondary care and reduce the burden on individual nurses.

For patients, there are also novel approaches to support a healthy lifestyle and weight loss. The emergence of anti‐obesity medications offers additional support for individuals striving to achieve weight loss goals (Chakhtoura et al. 2023). While these medications can be beneficial, it is essential to emphasise that a healthy lifestyle remains the cornerstone for sustainable weight management.

The use of eHealth to support patients in modifying their lifestyle is gaining increasing recognition (Duan et al. 2021). eHealth offers tools that empower patients to take more responsibility for their lifestyle, a belief also emphasised by diabetes nurses. eHealth provides patients with insights into their lifestyle and offers personalised feedback and coaching. This approach encourages proactive health engagement and supports self‐management (Vandelanotte et al. 2016). Additionally, the results highlight the importance of a patient‐centred approach. eHealth can play a supportive role in this by providing deeper insights into the patient's condition, thus enabling tailored advice to be delivered based on individual needs.

### 6.2 Strengths and Limitations of the Work

A strength of this study is its methodological foundation. The interview questions and prompts were derived from existing literature and the CFIR, providing a theoretical underpinning to our approach. For data analysis, we employed an inductive thematic analysis, independently conducted by two coders. The codes were compared and discussed until consensus was reached, adhering to the standards for qualitative data analysis (Braun and Clarke 2006).

One limitation of this study is its relatively small sample size, which may limit the generalizability of the findings. Due to the exploratory nature of this study, we adopted the concept of information power (Malterud et al. 2016). The aim was not to develop a comprehensive theory but rather to offer new insights that contribute meaningfully to the research questions. The study's clear focus and the depth of the interviews ensured that the collected perspectives were relevant and insightful. Additionally, a smaller sample size can still be sufficient when the data are rich and closely aligned with the study's objectives, particularly in exploratory research (Malterud et al. 2016; Guest et al. 2006). Thus, despite the limited number of participants, we were able to collect meaningful data.

### 6.3 Recommendations for Further Research

To enhance the generalizability of the findings, research across multiple hospitals is recommended. This would clarify the influence of setting‐specific factors, such as the presence of internal lifestyle programmes, on the outcomes. Involving medical specialists in future research is beneficial for a multidisciplinary approach and a broader perspective on lifestyle implementation.

### 6.4 Implications for Policy and Practice

The findings offer several practical implications for healthcare professionals and policymakers regarding the integration of lifestyle into T2DM care. First, specialised training in lifestyle guidance is essential for diabetes nurses to further develop their expertise. This will improve their ability to support and motivate patients to make lifestyle changes. Policymakers can support this by developing practical guidelines that align with daily clinical practice. Such guidelines should offer actionable steps and referral pathways for integrating lifestyle advice into routine consultations.

Furthermore, the study revealed that providing intensive lifestyle guidance is often beyond the scope of diabetes nurses' responsibilities. Therefore, promoting multidisciplinary collaboration is crucial. The involvement of lifestyle experts, such as dietitians and lifestyle coaches, might improve the effectiveness of lifestyle interventions both within hospitals and in broader regional networks.

Finally, the study highlights the potential of anti‐obesity medications and eHealth technologies to support lifestyle behaviours. Integrating these eHealth solutions into routine care could allow for more patient empowerment and personalised lifestyle management.

## 7 Conclusions

This study highlights that lifestyle is a recognised aspect in the consultations conducted by diabetes nurses in secondary care. However, several barriers make discussing lifestyle difficult, including limited expertise, the limited practicality of guidelines, time constraints, patient relationships, and low patient motivation. Diabetes nurses recognise the importance of addressing lifestyle in every consultation. However, they see their role mainly as raising awareness, providing education, and offering brief personalised advice rather than delivering extensive, individualised coaching. Nurses primarily focus on identifying patients' needs and referring them to appropriate professionals or interventions. To promote lifestyle discussions within consultations, there is a need for expertise development and practical guidelines. Advances in medicine and technology can empower and support individuals in achieving a healthy lifestyle and promoting weight loss. Diabetes nurses emphasise the importance of enhanced collaboration within hospitals and at regional level to facilitate a holistic approach to promoting lifestyle.

## Author Contributions

Conceptualisation, E.A.G.H. and G.D.L.; methodology, C.H.M.P. and E.A.G.H.; validation, C.H.M.P., E.A.G.H., and G.D.L.; formal analysis, C.H.M.P. and E.A.G.H.; investigation, C.H.M.P.; resources, C.H.M.P. and E.A.G.H.; data curation, C.H.M.P. and E.A.G.H.; writing – original draft preparation, C.H.M.P. and E.A.G.H.; writing – review and editing, C.H.M.P., E.A.G.H., and G.D.L.; visualisation, E.A.G.H.; supervision, C.H.M.P. and E.A.G.H.; project administration, C.H.M.P.; funding acquisition, G.D.L. All authors have read and agreed to the published version of the manuscript.

## Conflicts of Interest

The authors declare no conflicts of interest.

## Supporting information


Appendix S1.

## Data Availability

The data that support the findings of this study are available on request from the corresponding author. The data are not publicly available due to privacy or ethical restrictions.
