# Supplementary material for: Implementing Lifestyle Counselling Into Secondary Type 2 Diabetes Care: Perspectives From Diabetes Nurses
Source: Nurs Open. 2025 Apr 14;12(4):e70183. doi: 10.1002/nop2.70183 (PMC11995167; doi:10.1002/nop2.70183)
Supplement: Supplementary file 1 — Appendix S1. [file NOP2-12-e70183-s001.pdf]

**Supporting Information 1. Translated interview schedule**

1. **Introduction**

Hello,

Before we start with the interview, let me first introduce myself.

*<Introducing>*

This interview is part of a study on lifestyle discussions in consultations with type 2 diabetes patients. I would like to thank you in advance for participating in this interview.

As diabetes nurse, you see patients every three months, and the average duration of a consultation is around 30-45 minutes. These consultations are more comprehensive than those with the internist, which last for 10 minutes. Therefore, you play a crucial role in the care of people with type 2 diabetes. During this interview, I will inquire about your opinions on various aspects related to lifestyle in the consultation, such as how you currently discuss lifestyle, factors influencing the conversation, and your vision for the future regarding lifestyle in healthcare.

You have the opportunity to share your own opinions, and there are no right or wrong answers. If you do not know the answer to a question or feel uncomfortable providing a response, please indicate so. You have the right to refuse to answer questions you do not wish to respond to. You also have the right to withdraw from the study at any time. The answers will be pseudonymized and used solely for this research. With your consent, I would like to record this interview. Do you agree with all the aforementioned? If so, I kindly ask you to sign the informed consent form.

*<Signing informed consent>*

The interview will last approximately 30 to 45 minutes. Are there any questions before we begin the interview? If not, I will start the recording, and we will commence.

*<Start audio recording>*

## 2. Interview topics and questions

**Table A1.** Interview topics, questions/prompts and probes

<table>
<tr><th>Topic</th><th>Questions and prompts</th><th>Possible probes</th></tr>
<tr><td>Current situation</td><td>I would like to start with a general question.<ul><li>What does lifestyle mean to you?</li></ul>You treat people with type 2 diabetes daily in your consultations.<ul><li>What does a consultation with type 2 diabetes patients look like? (prompts: main tasks, topics discussed, duration, use of tools such as technology or conversation cards)</li><li>What is the role of lifestyle in your consultations? (prompts: which lifestyle domains, discussing current lifestyle, providing advice, referring to other healthcare professionals)</li></ul></td><td><ul><li>Why do you discuss these topics?</li><li>How do you address this?</li><li>Why do you use these guidelines/method?</li><li>Can you tell me more about that?</li></ul></td></tr>
<tr><td>Facilitating and hindering factors for lifestyle discussion</td><td>I would like to learn more about why you discuss lifestyle with patients in your consultation or not.<ul><li>What factors influence the conversation about lifestyle in people with type 2 diabetes?<br>Ask about facilitating and hindering factors for the individual domain (e.g., motivation, capability, patient-related factors), inner setting (e.g., work culture, available resources), and outer setting (e.g., policies and guidelines, financing).</li></ul></td><td><ul><li>How do you handle this?</li><li>What do you think about that?</li><li>Why are these areas for improvement?</li><li>How would you apply this?</li><li>How could this barrier be solved?</li></ul></td></tr>
</table>

| | | |
|---|---|---|
| Future vision | Additionally, I am curious about your opinion regarding the future role of lifestyle in healthcare.<br><br>• What is your vision regarding lifestyle in type 2 diabetes care in the future? (prompts: focus on lifestyle in consultations, role and task allocation among different healthcare professionals)<br>    o What is needed to achieve this?<br>    *Ask what is needed on the individual level (e.g., knowledge, skills), intervention/innovations (e.g., tools, technology), inner setting (e.g., education/training, time), and outer setting (e.g., guidelines)*<br><br>Nowadays, eHealth and other technology are becoming increasingly popular. This includes online consultations, sensors, online programs that patients can participate in, and health apps on smartphones.<br><br>• What do you think about the use of technology and eHealth to discuss or promote lifestyle in people with type 2 diabetes?<br>*Ask about expected benefits or challenges or prerequisites for integration* | • Why these specific points?<br>• What benefit does it provide you?<br>• How do you envision that?<br>• How should this be implemented? |

### 3. Conclusion

That concludes the interview. Do you have any questions or topics we have not discussed yet? I would like to thank you once again for your effort and time in participating in this interview for my research.

*<Stop recording>*
